# Single-Atom Site Photocatalysts Boosting Organic Synthesis: The Integration of a Metal Active Site and Photosensitive Unit

**DOI:** 10.3390/nano16020129

**Published:** 2026-01-19

**Authors:** Haoyue Sun, Yu Yang, Yanchang Liu, Dongxue Yang, Yichang Liu, Zaicheng Sun

**Affiliations:** 1 State Key Laboratory of Materials Low-Carbon Recycling, Center of Excellence for Environmental Safety and Biological Effects, Beijing Key Laboratory for Green Catalysis and Separation, Institute of Hydrogen Energy Daxing, Department of Chemistry, College of Chemistry and Life Science, Beijing University of Technology, Beijing 100124, China; shy-010203@emails.bjut.edu.cn (H.S.); yangyu@emails.bjut.edu.cn (Y.Y.); 2 Industrial and Information Industry Promotion Center, Inner Mongolia Autonomous Region, Chifeng 024005, China; jwzhk528@126.com

**Keywords:** metallaphotoredox catalysis, single-atom site catalysts, organic synthesis, structure–activity relationship

## Abstract

Metallaphotoredox catalysis merges the powerful bond-forming abilities of transition metal catalysis with unique electron or energy transfer pathways accessible in photoexcited states, injecting new vitality into organic synthesis. However, most transition metal catalysts cannot be excited by visible light. Thus, prevalent metallaphotoredox catalytic systems require dual catalysts: a transition metal catalyst and a separate photosensitizer. This leads to inefficient electron transfer between these two low-concentration catalytic species, which often limits overall photocatalytic performance. Single-atom site catalysts (SASCs) offer a promising solution, wherein isolated and quasi-homogeneous transition metal sites are anchored on heterogeneous supports. When semiconductors are employed as the support, the photosensitive unit and transition metal catalytic site can be integrated into one system. This integration switches the electron transfer mode from intermolecular to intramolecular, thereby significantly enhancing photocatalytic efficiency. Furthermore, such heterogeneous catalysts are easier to separate and reuse. This review summarizes recent advances in the application of SASCs for photocatalytic organic synthesis, with a particular focus on elucidating structure–activity relationships of the single-atom sites.

## 1. Introduction

**Figure 1:**
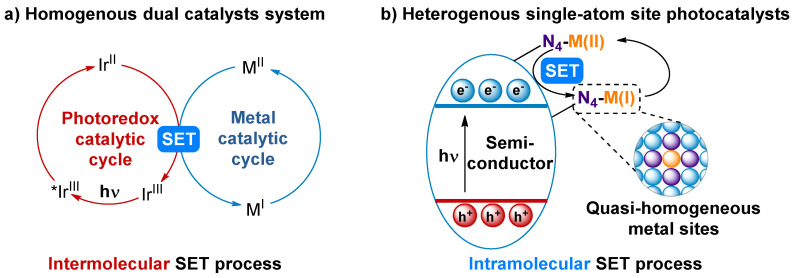
Metallaphotoredox catalysis. (**a**) Homogeneous system with dual catalysts. (**b**) Heterogeneous single-atom site photocatalysts.

In recent years, the development of milder, more eco-friendly catalytic systems for organic synthesis has emerged as a pivotal research priority in addressing the energy crisis [1,2]. Solar energy, as a clean and renewable resource, has propelled photochemical organic synthesis into a realm of widespread attention and rapid advancement [3,4,5]. Beyond using a greener energy source, the electron transfer pattern in photocatalytic processes differs fundamentally from that in ground-state chemistry [6,7,8]. Under light irradiation, excited-state photocatalysts activate substrates through single-electron transfer (SET) or energy transfer (ET). The alteration in the electron transfer mode brings novel reaction pathways and selectivity. To expand the application scope of photochemical organic synthesis, researchers have integrated transition metal catalysis with photoredox catalysis [9]. The photoredox process avoids the use of strong oxidants or reductants, improving the reaction’s functional group tolerance. On the other hand, single-electron redox processes offer novel strategies for the modulation of transition metal valence states. This combination effectively merges the strong bond-forming ability of transition metal catalysis with the unique electron transfer characteristics of photochemistry. However, most transition metal catalysts cannot be directly excited by visible light. Therefore, a dual-catalyst system, consisting of the transition metal catalyst and photosensitizer, is required (Figure 1a). The challenges of this system are the low efficiency of electron transfer between the two low-concentration catalytic species and increased reaction cost, due to the complex catalyst composition.

Since the first report in 2011, single-atom site catalysts (SASCs) have attracted widespread attention and been used in various fields [10,11]. In SASCs, quasi-homogeneous and isolated metal sites are anchored on the surface of heterogeneous supports. These single-atom sites have a first-shell coordination structure like that of homogeneous metal complexes, enabling them to facilitate organometallic elementary reactions for organic synthesis [12,13]. Thus, SASCs are regarded as a bridge between homogeneous and heterogeneous catalysis [14]. When the semiconductors, which have visible-light absorption, are used as supports and modified with single-atom sites, the single-atom site photocatalysts are obtained. In these catalysts, the photosensitive unit and the transition metal catalytic site are integrated into one. Compared with dual-catalyst systems, the electron transfer mode changes from intermolecular to intramolecular, significantly enhancing photocatalytic efficiency (Figure 1b). On the other hand, these ‘two-in-one’ heterogeneous catalysts are easier to separate and recycle.

This review summarizes the applications of SASCs in photochemical organic synthesis, categorized by the structure–activity relationships of single-atom sites: (1) catalyzing oxidative addition/reductive elimination processes; (2) promoting water splitting reaction combined with organic synthesis; and (3) regulating radical reactivity.

## 2. SASCs Catalyzing Oxidative Addition/Reductive Elimination Processes

Oxidative addition and reductive elimination are a pair of elementary reactions in organometallic chemistry, and represent one of the most common mechanistic pathways for cross-coupling reactions. Well-known cross-coupling reactions, such as the Suzuki–Miyaura reaction, Negishi reaction, Buchwald–Hartwig reaction, and Ullmann reaction, rely on the oxidative addition and reductive elimination to form C-C or C-X bonds. Low-valent transition metals facilitate the oxidative addition process. However, excessively rapid reduction may reduce the transition metal to its zero-valent state, leading to aggregation and deactivation. In single-atom photocatalysis, light irradiation provides mild reduction conditions, while isolated metal sites prevent the aggregation of low-valent metals.

### 2.1. Ni Single-Atom Site Photocatalysts

Carbon nitride is a “star material” with good thermal stability, low toxicity, a suitable band structure, and low cost [15,16]. Notably, the N-heterocyclic structures in its skeleton are ideal ligands for constructing Ni-N_x_ sites.

**Figure 2:**
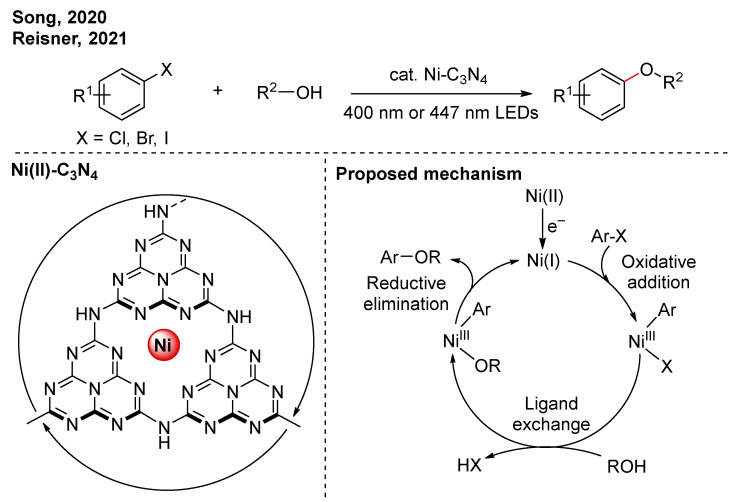
Ni-C_3_N_4_ photocatalytic C-O cross-coupling reactions [17,18].

In 2020, Song and co-workers constructed Ni(II) single-atom sites on the carbon nitride (Ni-C_3_N_4_) (Figure 2) [17]. Under light irradiation, the photo-induced electron could reduce the Ni(II) to Ni(I), which promotes the oxidative addition. Through the oxidative addition, ligand exchange, and reductive elimination steps, C-O cross-coupling between aryl halides and alcohols is achieved via a Ni(I)-Ni(III)-Ni(I) catalytic cycle. And the alcohol also acts as a hole scavenger. Comparing previous reports, this approach avoids the use of Ru- or Ir-based photocatalysts. Reisner’s group reported a similar catalytic system that is compatible with aryl chlorides [18].

**Figure 3:**
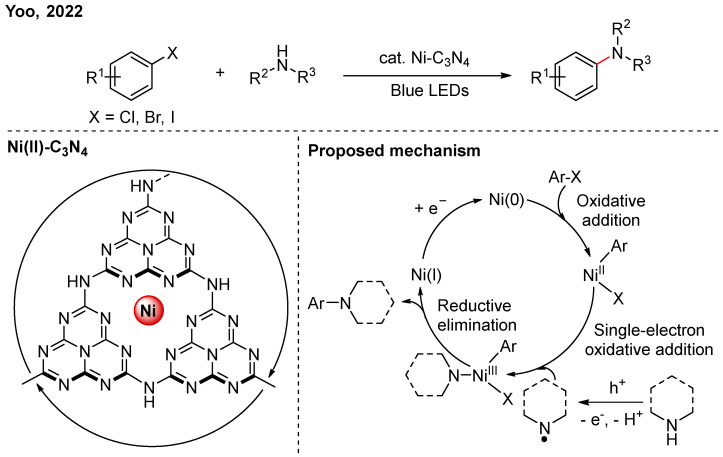
Ni-C_3_N_4_ photocatalytic C-N cross-coupling reactions [19].

When amines are used as nucleophiles instead of the alcohols, the catalytic mechanism changes. Yoo and co-workers have realized the C-N cross-coupling using the Ni-C_3_N_4_ photocatalyst (Figure 3) [19]. Due to the low oxidation potential of amines, they are oxidized by holes to generate *N*-centered radicals. These radicals subsequently undergo single-electron oxidative addition with Ni(II). Throughout the entire catalytic cycle, the C-N bond is formed via oxidative addition, single-electron oxidative addition, and reductive elimination steps. The catalytic cycle of Ni shows as Ni(0)-Ni(II)-Ni(III)-Ni(I)-Ni(0).

**Figure 4:**
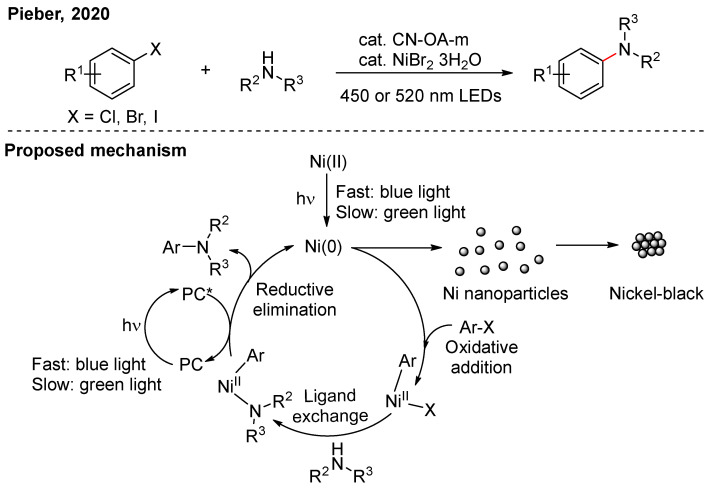
The aggregation of Ni(0) under light irradiation [20].

In the above C-N cross-coupling, electron-rich aryl halides lead to low yields. The reason is that the slow oxidative addition step causes Ni(0) to aggregate into nanoparticles, deactivating the catalyst. To address this issue, Pieber’s group replaced blue light with longer-wavelength green light (Figure 4) [20]. This modification reduces the rates of both photoinduced reductive elimination and the reduction in Ni(II) to Ni(0), significantly improving the catalyst’s cycling stability. High-angle annular dark field scanning transmission electron microscopy (HAADF-STEM) images showed that distinct Ni nanoparticles were observed under blue light irradiation, whereas no aggregation was observed under green light.

**Figure 5:**
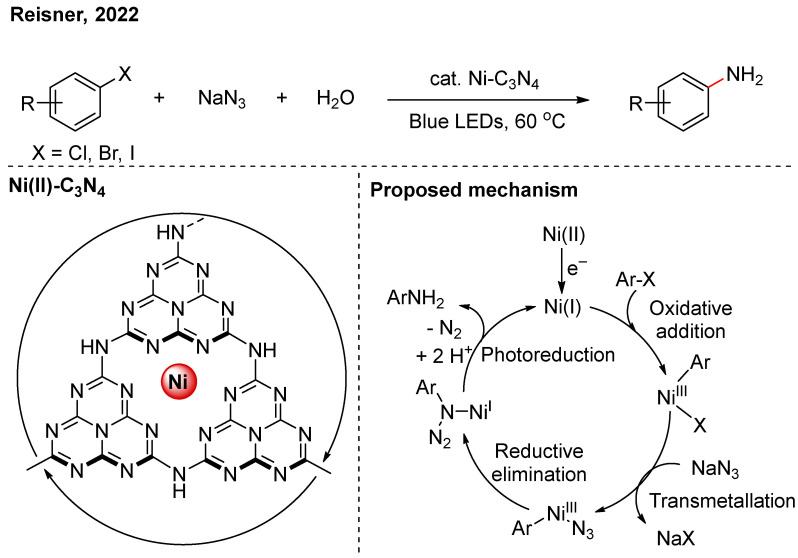
Ni-C_3_N_4_ photocatalytic amination of aryl halides [21].

In 2022, Reisner used sodium azide as the nucleophile in the Ni-C_3_N_4_ photocatalytic system (Figure 5) [21]. Transmetalation between the sodium azide and Ni(III) forms a Ni(III)-N_3_ species. Subsequent reductive elimination produces Ar-N_3_, which coordinates with Ni(I). Photoreduction and denitrogenation then yield the corresponding aniline, with H_2_O serving as the H donor.

**Figure 6:**
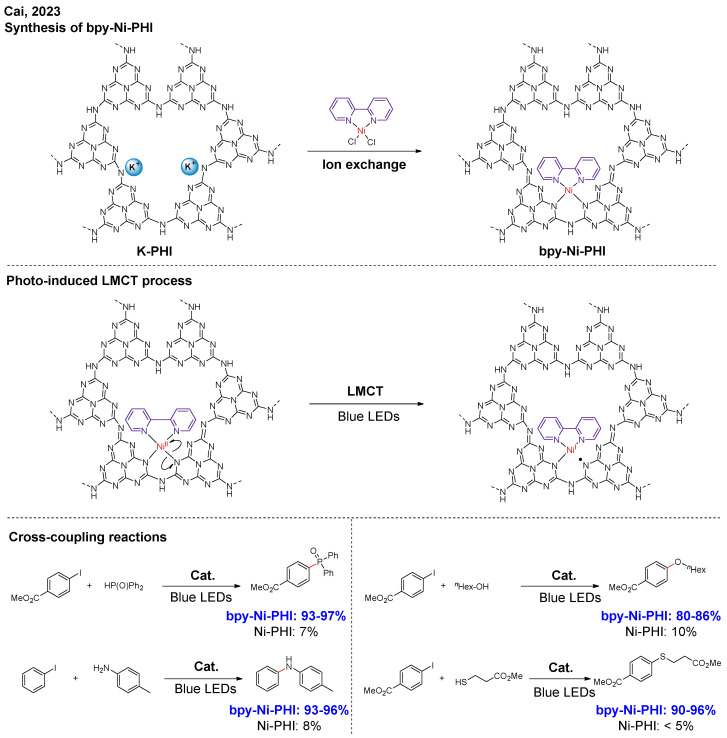
Homogeneous ligands modified Ni single-atom sites [22].

The ligands regulate the electronic and geometric properties of single-atom sites, playing an important role in enhancing catalytic reactivity. In the aforementioned works, Ni sites were coordinated with the carbon nitride, which serves as a heterogeneous ligand with an ambiguous structure. To precisely control the coordination environment, Cai and co-workers proposed modifying Ni single-atom sites with homogeneous ligands (Figure 6) [22]. Firstly, they synthesized crystalline potassium poly(heptazine imide) (K-PHI), and then introduced Ni sites via an ion-exchange process between a homogeneous Ni complex and K-PHI. A similar coordination environment around Ni sites could be retained. In various cross-coupling reactions, such as C-O, C-N, C-P, and C-S bond formation, bpy-Ni-PHI exhibited significantly improved catalytic performance compared to Ni-PHI. At bpy-Ni-N_2_ sites, the ligand to metal charge transfer (LMCT) process occurs under blue LED irradiation, producing Ni(I) species and *N*-centered radical. The dipyridyl (bpy) ligands facilitate this LMCT process, increasing the concentration of Ni(I) species, which, in turn, promotes the reaction.

### 2.2. Pd Single-Atom Site Photocatalysts

**Figure 7:**
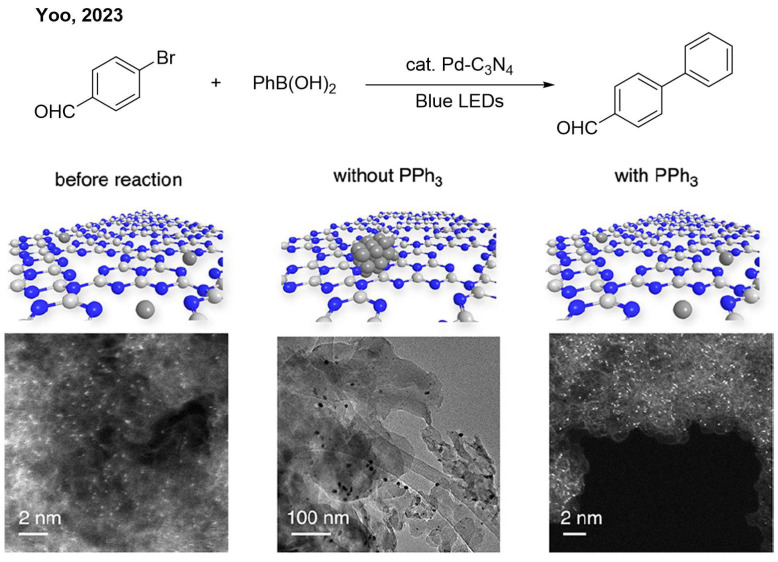
The aggregation of Pd(0) under light irradiation. Reproduced with permission from [23], Copyright 2023, American Chemical Society.

Like nickel, palladium can also be reduced to the zero-valent state by photogenerated electrons, favoring oxidative addition. The Suzuki–Miyaura reaction is one of the most classical and important reactions involving the oxidative addition process. Yoo and co-workers anchored Pd single-atom sites on the surface of carbon nitride (Figure 7) [23]. The photo-generated electrons from C_3_N_4_ transfer to Pd sites, reducing them to Pd(0), which is the active intermediate for the Suzuki reaction. However, the Pd nanoparticles could be detected after the reaction, reducing the catalyst’s stability and recyclability. The author found that adding PPh_3_ as the ligand prevents the aggregation of Pd(0).

## 3. SASCs Catalyzed Water Splitting Combining with Organic Synthesis

Photocatalytic water splitting is a research hotspot in single-atom catalysis [24,25]. The water splitting can be divided into two half-reactions: oxygen evolution reaction (OER) and hydrogen evolution reaction (HER). Integrating these reactions with organic synthesis offers greener and more efficient synthetic pathways.

### 3.1. Combining Oxygen Evolution Reaction with Reductive Coupling

OER (produces O_2_ from water) is often considered economically less valuable. However, when water is viewed as a terminal reductant, electrons generated from water oxidation can drive reductive coupling reactions, providing a greener approach.

**Figure 8:**
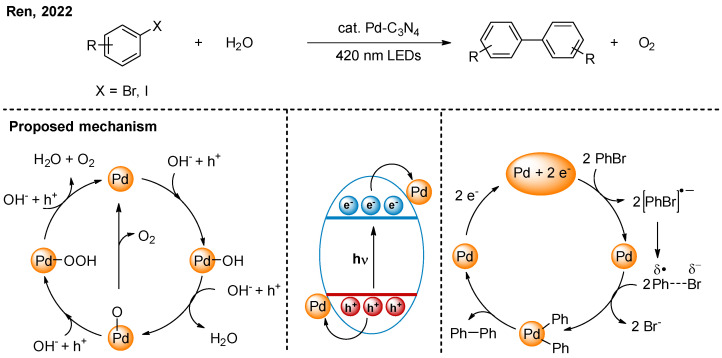
Pd-C_3_N_4_ photocatalytic reductive coupling of aryl halides with oxygen evolution reaction [26].

In 2022, Ren and co-workers combined the oxidative half-reaction of water with reductive homo-coupling of aryl halides (Figure 8) [26]. Under light irradiation, Pd single-atom sites both accept electrons from the conduction band of carbon nitride and donate electrons to the hole. On the reduction side, Pd can further transfer electrons to aryl halides, reducing them to aryl radicals. Subsequently, two aryl radicals can combine with Pd to form a C-Pd-C species. Finally, C-C coupling products are obtained via reductive elimination. On the oxidation side, the four-electron one-step process has been proven as the major pathway. Pd is oxidized and continuously reacts with -OH, ultimately releasing the oxygen.

**Figure 9:**
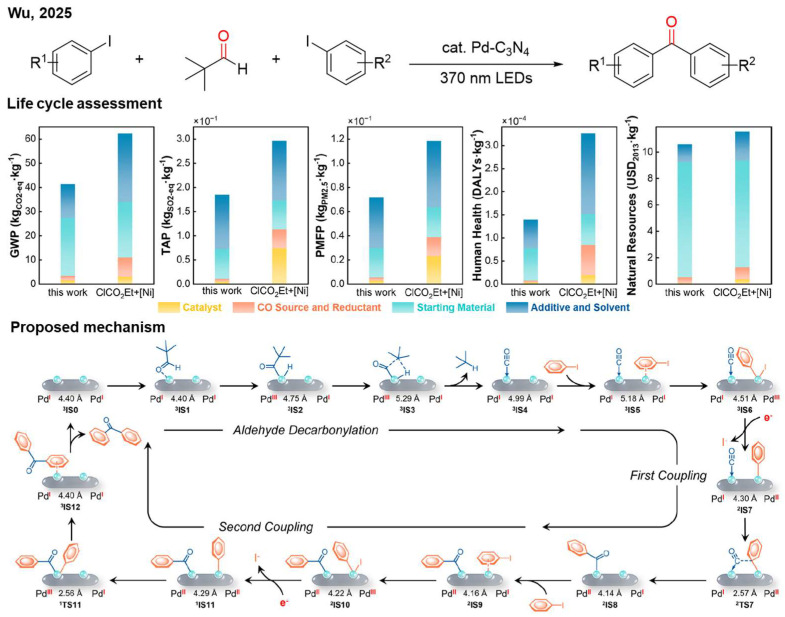
Pd-C_3_N_4_ photocatalytic reductive carbonylation with oxygen evolution reaction. Reproduced with permission from [27], Copyright 2025, American Chemical Society.

Later, Wu and co-workers introduced pivaldehyde as the carbonyl source to achieve reductive carbonylative coupling (Figure 9) [27]. Two molecules of iodobenzene and pivaldehyde were used to synthesize the benzophenone. The heterogeneous Pd single-atom site photocatalyst is compatible with continuous flow systems and exhibits good cycling stability, demonstrating potential for scaled-up production. A life cycle assessment (LCA) highlights the notable environmental advantages of this strategy, attributed to the use of water as a green reductant, inexpensive carbonyl sources, and recyclable catalysts. The proposed mechanism involves cooperative catalysis by dual Pd sites. (1) Photogenerated electrons reduce Pd to Pd(I); (2) pivaldehyde undergoes oxidative addition with Pd(I), releasing an alkane to form a Pd-CO species; (3) a neighboring Pd site is also reduced to Pd(I) and undergoes oxidative addition with iodobenzene; (4) carbonyl insertion forms an Ar-C(O)-Pd species; and (5) oxidative addition of another iodobenzene with Pd(I) and reductive elimination complete the second coupling.

### 3.2. Combining Hydrogen Evolution Reaction with Oxidative Coupling

Integrating HER with oxidative coupling is a “two-birds-with-one-stone” strategy with two key advantages: (1) oxidative reaction can generate value-added products, reducing the cost of hydrogen generation; and (2) protons serve as ideal and green oxidants.

**Figure 10:**
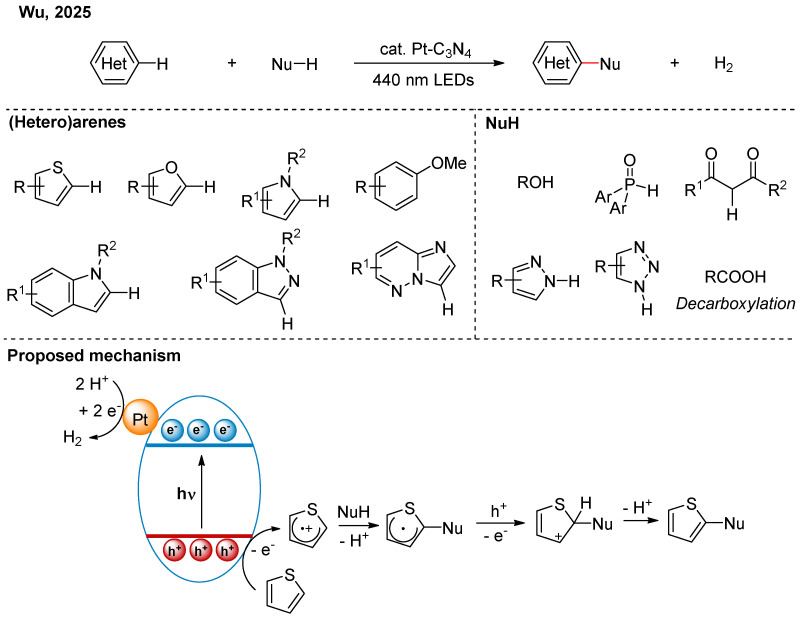
Pt-C_3_N_4_ photocatalytic CDC reactions with hydrogen evolution reaction [28].

C-H and heteroatom-H (X-H) compounds widely exist in nature. Through cross-dehydrogenative coupling (CDC) reactions, C-C and C-X bonds could be constructed directly using C-H and X-H compounds, which offers excellent step economy. However, traditional CDC reactions require stoichiometric oxidants, generating significant waste. In 2025, Wu and co-workers employed Pt–C_3_N_4_ as a photocatalyst to achieve CDC reactions between electron-rich (hetero)arenes and various nucleophiles, and H_2_ is the sole by-product (Figure 10) [28]. Mechanistic studies indicated that photogenerated holes can oxidize electron-rich (hetero)arenes to the corresponding radical cations, which are then attacked by nucleophiles. Subsequent oxidative aromatization yields the target product. Pt single-atom sites accept photogenerated electrons and transfer electrons to protons, releasing H_2_. The single-atom catalyst exhibits good cycling stability, low metal leaching, and a high turnover number, all of which outperform previous homogeneous catalytic systems.

**Figure 11:**
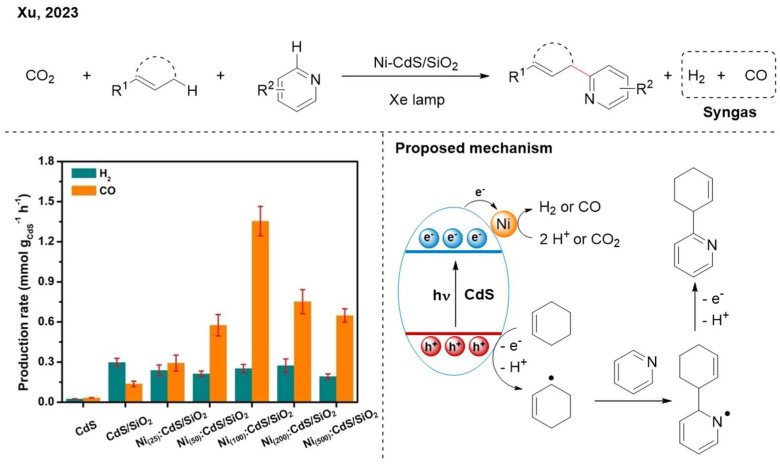
Ni-CdS/SiO_2_ photocatalytic C-C cross-coupling with CO_2_ reduction. Reproduced with permission from [29], Copyright 2025, Wiley.

Xu and colleagues introduced CO_2_ into an oxidative dehydrogenative cross-coupling reaction, producing both C-C coupling products and syngas (CO + H_2_) (Figure 11) [29]. They modified SiO_2_ with CdS quantum dots and anchored Ni single-atom sites onto the CdS. Notably, by adjusting the loading amount of Ni, the CO/H_2_ ratio can be turned from 1:2 to 5:1, encompassing the optimal syngas ratios required for methanol synthesis or Fischer–Tropsch synthesis. The reaction mechanism involves the oxidation adjacent to C=C bonds to generate *C*-centered radicals, which could be realized by the photogenerated holes on the CdS. Subsequently, the Minisci-type reaction between *C*-centered radicals and electron-deficient arenes can build the C-C bonds. Meanwhile, the nickel single atoms were responsible for reducing protons and CO_2_.

### 3.3. Photocatalytic Hydrogenation

When using water as the hydrogen source, some M-H species (such as Pd-H) exhibit a relatively slow rate of hydrogen evolution. In such cases, M–H intermediate can insert into unsaturated bonds, enabling hydrogenation. Integrating water activation and hydrogenation with suitable single-atom photocatalysts avoids high-concentration hydrogen gas, mitigating safety risks.

**Figure 12:**
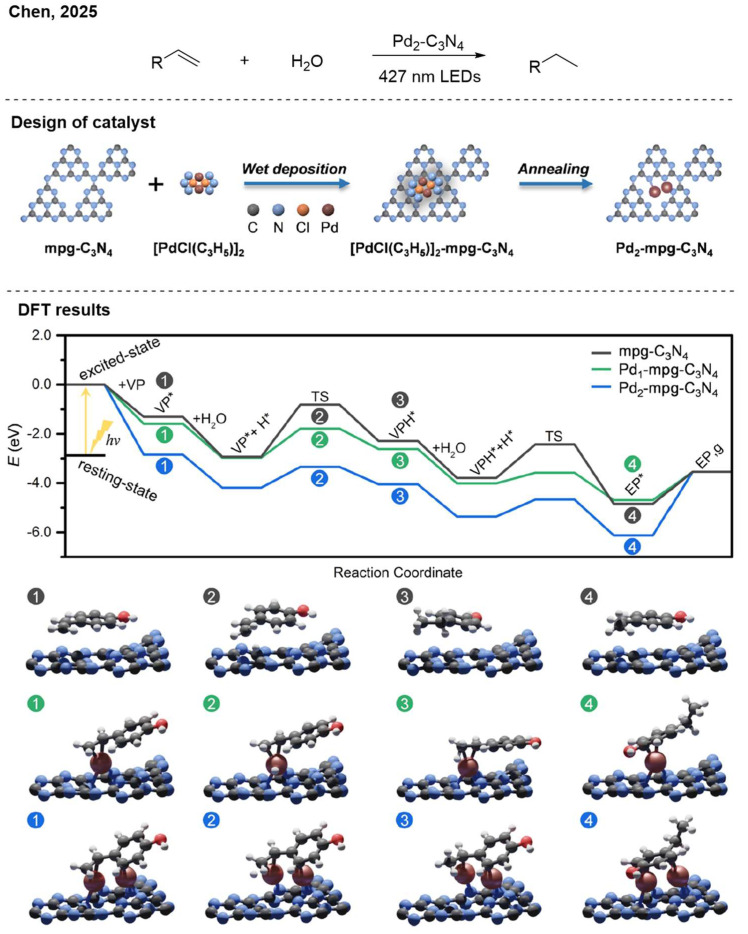
Pd_2_-C_3_N_4_ photocatalytic hydrogenation of alkenes. Reproduced with permission from [30], Copyright 2025, American Chemical Society.

In 2025, Chen et al. constructed diatomic Pd sites on the carbon nitride. Using water as the hydrogen source, they achieved the photocatalytic hydrogenation of various alkenes (Figure 12) [30]. In this system, the dual-atom catalyst demonstrated significantly higher efficiency compared to its single-atom counterpart. The density functional theory (DFT) simulations showed that the geminal Pd sites could enhance substrate adsorption while suppressing side HER.

**Figure 13:**
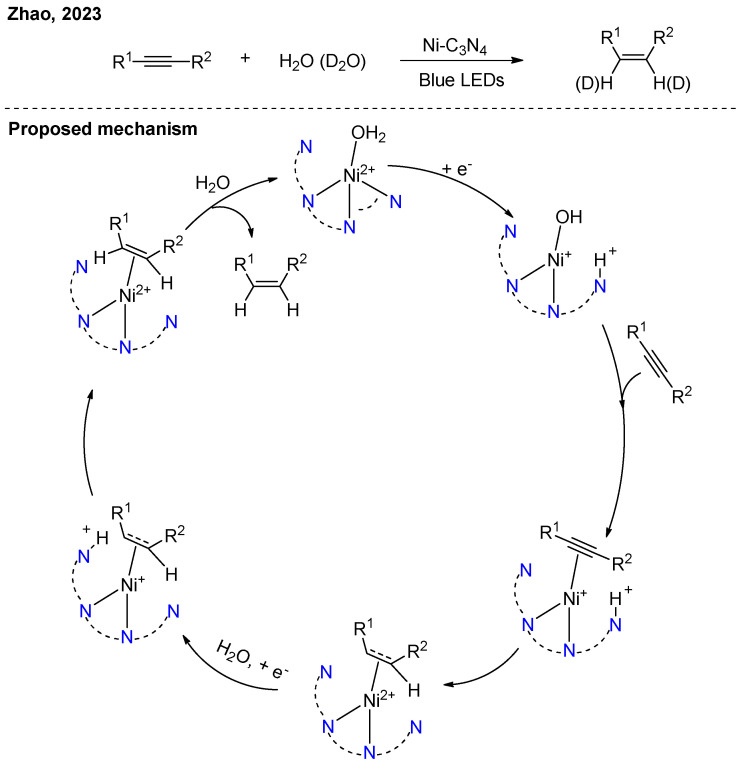
Ni-C_3_N_4_ photocatalytic semi-hydrogenation of alkynes [31].

By modifying the metal sites to Ni-N_4_, Zhao achieved selective semi-hydrogenation of alkynes (Figure 13) [31]. Photogenerated electrons can reduce nickel to Ni(I), while simultaneously forming an adjacent pyridinic N-H+ active site. Upon coordination of the alkyne to Ni(I), concurrent proton and electron transfer to the alkyne led to its hydrogenation. Due to the difference in adsorption affinity between alkenes and alkynes on the Ni species, this system achieved high chemoselectivity, preventing over-hydrogenation to alkanes. Notably, when D_2_O was used in place of water, deuterated alkenes can be synthesized.

Besides symmetric C=C bonds, the C=N double bond in imines can also be reduced. Using a Ru-CdS single-atom photocatalyst, Chen synthesized amino acids from lactic acid and aqueous ammonia [32]. In this process, photogenerated holes oxidized lactic acid to pyruvic acid. Subsequently, aqueous ammonia undergoes a condensation reaction with the carbonyl group to form an imine. The imine is finally reduced, which was promoted by the single-atom Ru species.

## 4. SASCs Regulating the Reactivity of Radicals

In photocatalysis, single-electron transfer (SET) is one of the primary reaction pathways. Consequently, radicals often serve as key intermediates. However, the vast majority of radicals have short half-lives, making it challenging to tame radicals. In homogeneous catalysis, transition metals can coordinate with radicals or undergo single-electron oxidative addition, which could regulate the activity of radicals and facilitate subsequent reactions. Similarly, in single-atom catalytic systems, metal sites can achieve the same effect.

**Figure 14:**
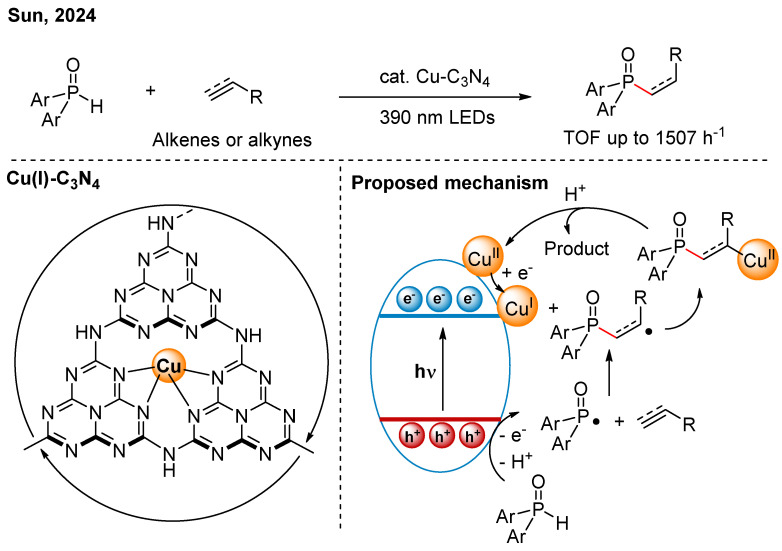
Cu-C_3_N_4_ photocatalytic hydrophosphonylation reactions of unsaturated hydrocarbons [33].

In 2024, Sun and co-workers reported that under photocatalytic conditions, the Cu-C_3_N_4_ could significantly enhance the efficiency of hydrophosphonylation reactions of unsaturated hydrocarbons (Figure 14) [33]. Mechanistic studies indicated that photogenerated holes can oxidize diphenylphosphine oxide to generate *P*-centered radicals. The phosphonyl radical then added to unsaturated hydrocarbons, forming corresponding *C*-centered radicals. The Cu(I)-N_4_ single-atom sites underwent single-electron oxidative addition with the *C*-centered radicals, forming Cu(II)-C species. This process could enhance the stability of the carbon radicals, thereby improving the reaction efficiency. Compared to previous reports, this system achieves a broader substrate scope and a higher turnover frequency.

**Figure 15:**
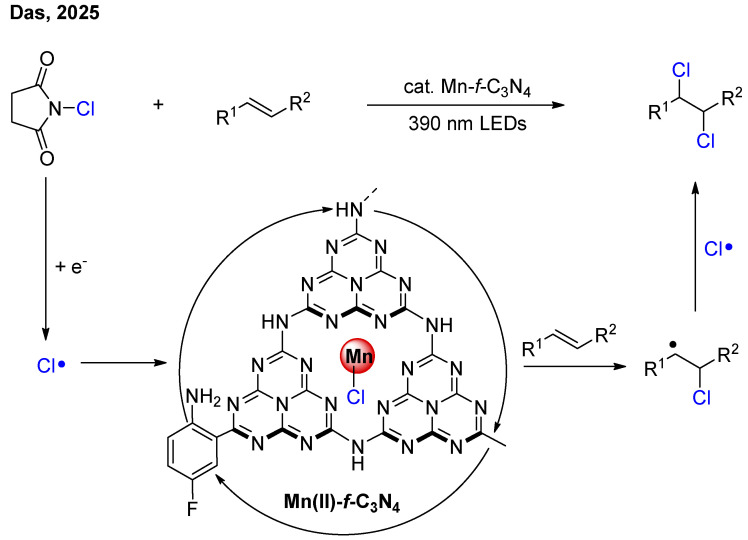
Mn-*f*-C_3_N_4_ photocatalytic dichlorination of alkenes [34].

In Das’s work, the Mn-C_3_N_4_ was used as a photocatalyst to achieve the dichlorination of alkenes (Figure 15) [34]. Through the redox capability of the carbon nitride, this system was compatible with a mild chlorination reagent, *N*-chlorosuccinimide. Notably, the Mn(II) single-atom sites could form Mn-Cl bonds with Cl radicals, thereby stabilizing the Cl radicals. Furthermore, by introducing a 2-amino-5-fluorophenyl group onto the carbon nitride framework, the visible-light absorption range of carbon nitride was enhanced.

## 5. Summary Table

In Table 1, we summarize the structures of single-atom sites, types of supports, and categories of organic reactions from all previous reports discussed in this review. In terms of metal types, Pd and Ni dominate, which facilitates oxidative addition and reductive elimination processes. For semiconductor supports, g-C_3_N_4_ is predominantly selected. The N atoms in g-C_3_N_4_ are regarded as ideal ligating atoms for anchoring transition metals. And cross-coupling reactions and hydrogenation are the primary organic reaction types in single-atom photocatalytic protocols.

## 6. Conclusions and Perspectives

This review summarizes recent advances in the application of single-atom site photocatalysts (SASCs) for organic synthesis, focusing on their structure–activity relationships. SASCs integrate photosensitive units and transition metal catalytic sites, overcoming the limitations of traditional dual-catalyst metallaphotoredox systems—such as inefficient electron transfer and high cost.

In oxidative addition/reductive elimination processes, Ni and Pd single-atom sites anchored on carbon nitride or modified with homogeneous ligands exhibit high activity and stability for cross-coupling reactions. In water splitting combined with organic synthesis, SASCs enable the integration of OER/HER with reductive/oxidative coupling or hydrogenation, providing greener synthetic pathways with water as a renewable resource. Additionally, SASCs show great potential in regulating radical reactivity, though further research is needed to fully explore this area.

Future directions for SASCs’ development include the following:

(1) Precise control of the coordination environment to optimize catalytic performance. In most previous reports of SASCs, only the first coordination shell of metal sites could be controlled precisely, while the outer-sphere is always ill-defined. This greatly restricts the types of organic synthesis reactions that can be effectively adapted. For certain reactions that rely on precise coordination structures, such as asymmetric catalysis, effective implementation remains unachievable. Therefore, developing new strategies to realize the precise regulation of the coordination environment is necessary.
(2) Expanding the scope of applicable organic reactions. Currently, almost all single-atom photocatalytic organic reactions can be achieved using homogeneous catalytic systems. Although heterogeneous catalysts offer advantages in terms of recovery and reusability, we still hope to see the unique structure of SASCs enabling novel reaction patterns, particularly by expanding beyond the scope of homogeneous catalysis.
(3) Enhancing catalyst stability and recyclability for industrial applications. The stability of SASCs remains unsatisfactory. During reactions, metal atoms are still prone to agglomeration or leaching into the solvent, which hinders further industrial application. On the other hand, scalable setups compatible with SASCs, such as flow photochemical setups, deserve to be developed.
(4) Deepening the understanding of reaction mechanisms through advanced characterization techniques and theoretical calculations. In homogeneous catalysis, organometallic chemistry has already been well developed. Establishing the connection between homogeneous catalysis and single-atom site catalysis can provide a novel perspective for understanding the mechanism and aid in designing new reaction pathways.

## Figures and Tables

**Table 1:** Some recent reported single-atom site photocatalysts in organic synthesis.

Single-Atom Sites	Support	Reactions	Refs.
Ni-N_x_	C_3_N_4_	C-O cross-couplings between ArX and alcohols	[17,18]
Ni-N_x_	C_3_N_4_	C-N cross-couplings between ArX and amines	[19,20]
Ni-N_x_	C_3_N_4_	C-N cross-couplings between ArX and NaN_3_	[21]
Ni-(bpy)N_2_	C_3_N_4_	C-P/C-N/C-O/C-S cross-couplings	[22]
Pd-N_x_	C_3_N_4_	Suzuki–Miyaura reaction	[23]
Pd-N_x_	C_3_N_4_	Reductive coupling of aryl halides with OER	[26]
Pd-N_4_	C_3_N_4_	Reductive carbonylation with OER	[27]
Pt-N_3_	C_3_N_4_	CDC reactions with HER	[28]
Ni-O_4_	CdS/SiO_2_	Minisci reactions with CO_2_ reduction and HER	[29]
Pd_2_-N_x_	C_3_N_4_	Hydrogenation of alkenes	[30]
Ni-N_4_	C_3_N_4_	Semi-hydrogenation of alkynes	[31]
Ru-S_x_	CdS	Synthesis of amino acids from lactic acid	[32]
Cu-N_4_	C_3_N_4_	Hydrophosphonylation reactions	[33]
Mn-N_6_	*f*-C_3_N_4_	Dichlorination of alkenes	[34]

## Data Availability

No new data were created or analyzed in this study. Data sharing is not applicable to this article.

## Abbreviations

The following abbreviations are used in this manuscript:

SASCs	Single-atom site catalysts
SET	Single-electron transfer
ET	Energy transfer
HER	Hydrogen evolution reaction
OER	Oxygen evolution reaction
